# Raising mountains for a habitable Earth

**DOI:** 10.1126/sciadv.aei2764

**Published:** 2026-11-13

**Authors:** Matthijs A. Smit, Douwe J. J. van Hinsbergen, Carl Guilmette, Ellen Kooijman, Amberlee V. King, Jamie A. Cutts, Wouter Bleeker

**Affiliations:** ^1^ Department of Earth, Ocean and Atmospheric Sciences, University of British Columbia, 2020-2207 Main Mall, Vancouver V6T 1Z4, British Columbia, Canada.; ^2^ Department of Geosciences, Swedish Museum of Natural History, Frescativägen 40, SE-114 18 Stockholm, Sweden.; ^3^ Department of Geosciences, Utrecht University, Princetonlaan 8a, NL-3584 CB Utrecht, Netherlands.; ^4^ Département de Géologie et Génie Géologique, Université Laval, 1045 avenue de la Méde-cine, Québec City G1V 0A6, Canada.; ^5^ Natural Resources Canada, Geological Survey of Canada, 601 Booth Street, Ottawa K1A 0G1, Ontario, Canada.

## Abstract

The formation of mountain belts (orogens) elevates rocks above sea level, making them available for chemical weathering and altering the composition of the atmosphere and oceans, which in turn control habitability. Peak-metamorphic pressures recorded by crustal rocks buried in orogens increased between 2.7 to 2.2 and 0.8 to 0.7 billion years ago, indicating that rocks could be buried deeper and that orogens could grow substantially thicker after each of these periods. The two periods of orogenic growth were broadly synchronous with major increases in terrigenous element and nutrient fluxes and changes in biological sulfur metabolism and surface oxygenation. These changes could be explained by enhanced weathering of higher mountains. The findings support the hypothesis that there are first-order mechanistic links between secular geodynamic change and changes in Earth system state and habitability, which shaped the conditions for the evolution of complex life during the past 3 billion years.

## INTRODUCTION

Continental silicate weathering controls the oceanward flux of carbon and bioactive nutrients that limit marine primary productivity, thus exerting control on atmospheric chemistry, climate, and marine biomass and biodiversity (*1*, *2*). The majority of the global terrigenous runoff derives from mountainous regions (*3*). On a regional scale and in the short term, the mass flux from such regions is a function of mean surface temperature, humidity, relief-runoff relationships, rates of topographic decay, weatherability, and other orogen-specific factors (*4*, *5*). On a global scale and in the long term, the time-integrated mass flux of sediment from the continents to the oceans is coupled to the prevalence and maximum elevation of mountain ranges. This sediment yield is controlled by erosion above the 3-km altitude (*3*, *6*) and scales exponentially with mean drainage-basin elevation (*4*). Terrain elevation in mountain belts relative to the mean sea level is controlled by the shear stress on plate contacts and relates to the thickness and density of the orogenic crust across tectonic settings (*7*, *8*). All of these factors have changed with time. Between 3.2 and 2.5 billion years (Gyr) ago, the surface area of the continental crust increased, the composition of the crust changed, and the crust emerged more prominently above sea level (*9*–*13*). Substantial amounts of seawater were gradually being recycled back into the mantle as the global geodynamic regime shifted toward plate tectonics moderated by wholesale subduction of oceanic lithosphere (*14*–*17*). Various pan-continental landmasses have been assembled and broken up in the wake of this shift (*18*–*20*), leaving the modern continents as complex amalgamations of variably differentiated crustal fragments.

The changes to the solid Earth form the backdrop to the many changes that the Earth system state has gone through since the hot, anoxic, and lifeless conditions of the early Archaean. The most pivotal among these changes are as follows: (i) the broadly two-stage rise in the flux of elements and bioactive nutrients to the oceans and associated rises in biological sulfur (S) metabolism and primary productivity (*21*–*23*); (ii) two major atmospheric oxidation events: the Great Oxidation Event (GOE; 2.4 to 2.2 Gyr ago) culminating from the stepwise oxygenation of the oceans since 3.0 Gyr ago and the Neoproterozoic Oxidation Event (NOE; 0.8 to 0.6 Gyr ago), which appears to be linked to increased primary productivity from photosynthesizing cyanobacteria and faster burial of organic carbon at the time (*24*–*29*); (iii) two “Snowball Earth” icehouse episodes, each one occurring in the wake of one of the above oxidation events (*29*–*32*); and (iv) the origin and diversification of complex life and associated growth of global biomass (Fig. 1; see Supplementary Text for context) (*33*). Broad temporal consistencies among these changes have inspired a myriad of propositions regarding the complex feedback and forcing mechanisms that may have brought about these staggering changes (Supplementary Text). Links to the evolving tectonic regime and orogenic processes are suspected (*9*, *15*) but are still enigmatic.

**Fig. 1.**
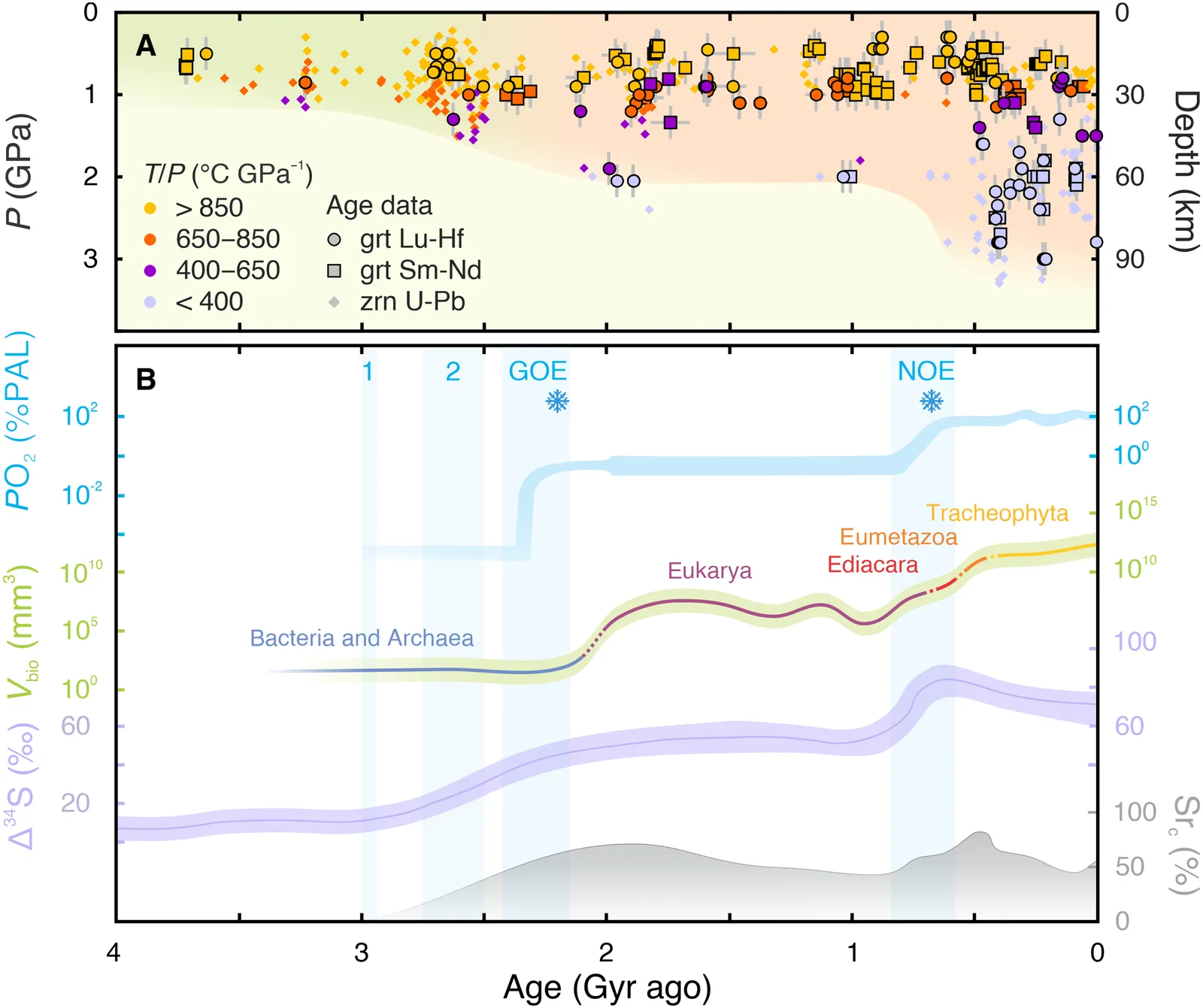
Metamorphic record in the context of Earth system change. (**A**) Peak-*P* conditions of continental metamorphic rocks through time. The envelope was constructed by extrapolating smoothly between prevalent maxima and provides an interpretive framework for secular metamorphic change, as reflected by the *P* data. Depths as indicated by the *P* condition assuming lithostatic conditions are shown for reference. Colors indicate episodes during which the continental crust was andesitic (orange) or more mafic (green) (*62*, *63*). (**B**) Evolution of atmospheric *P*o_2_ relative to the present atmospheric levels (PAL) (*28*), the biovolume (*33*), S isotope diversity in marine pyrite as a measure of biological S metabolism [Δ^34^S is the difference between the highest and lowest δ^34^S values observed for pyrite at a given age; determined from δ^34^S records; (*22*)], the percentage of oceanic Sr that is continent-derived [Sr_c_; (*21*)]. Oxygenation stages are shown for reference, marking early oxidative weathering (1), early “whiffs” of oxygenation and the rise of organisms capable of oxygenic photosynthesis [2; (*28*)], the GOE (*29*), and the NOE (*27*). Snowflake symbols indicate main icehouse stages [Huronian Glaciation at ∼2.2 Gyr ago and the Sturtian and Marinoan Glaciations at 717 to 659 and 650 to 635 Myr ago (*29*, *32*)]. For further context, see Supplementary Text.

Answers to pertinent questions regarding causation in this context require a time-resolved record of crustal thickness in mountainous regions. There is nevertheless substantial difficulty in reconstructing the paleo-depth of the crust-mantle boundary (Mohorovičić discontinuity or “Moho”). Bulk-rock chemical indices for Moho depth in igneous rocks [e.g., strontium (Sr)/yttrium (Y) and lanthanum (La)/ytterbium (Yb)] may work well in modern arcs (*34*) but may not be reliable in non-arc settings. Other chemical approaches, such as zircon Eu (europium) anomalies and other rare earth element–based indices, on the other hand, depend too strongly on case-specific parameters to enable their use in the investigation of global phenomena and secular change (*35*). As an alternative, constraints may be obtained through the metamorphic rock record (Fig. 2 and Materials and Methods), which ultimately reflects the depths to which rocks in orogens were buried and metamorphosed. In this study, we use this record to investigate the secular evolution of orogenic thickness.

**Fig. 2.**
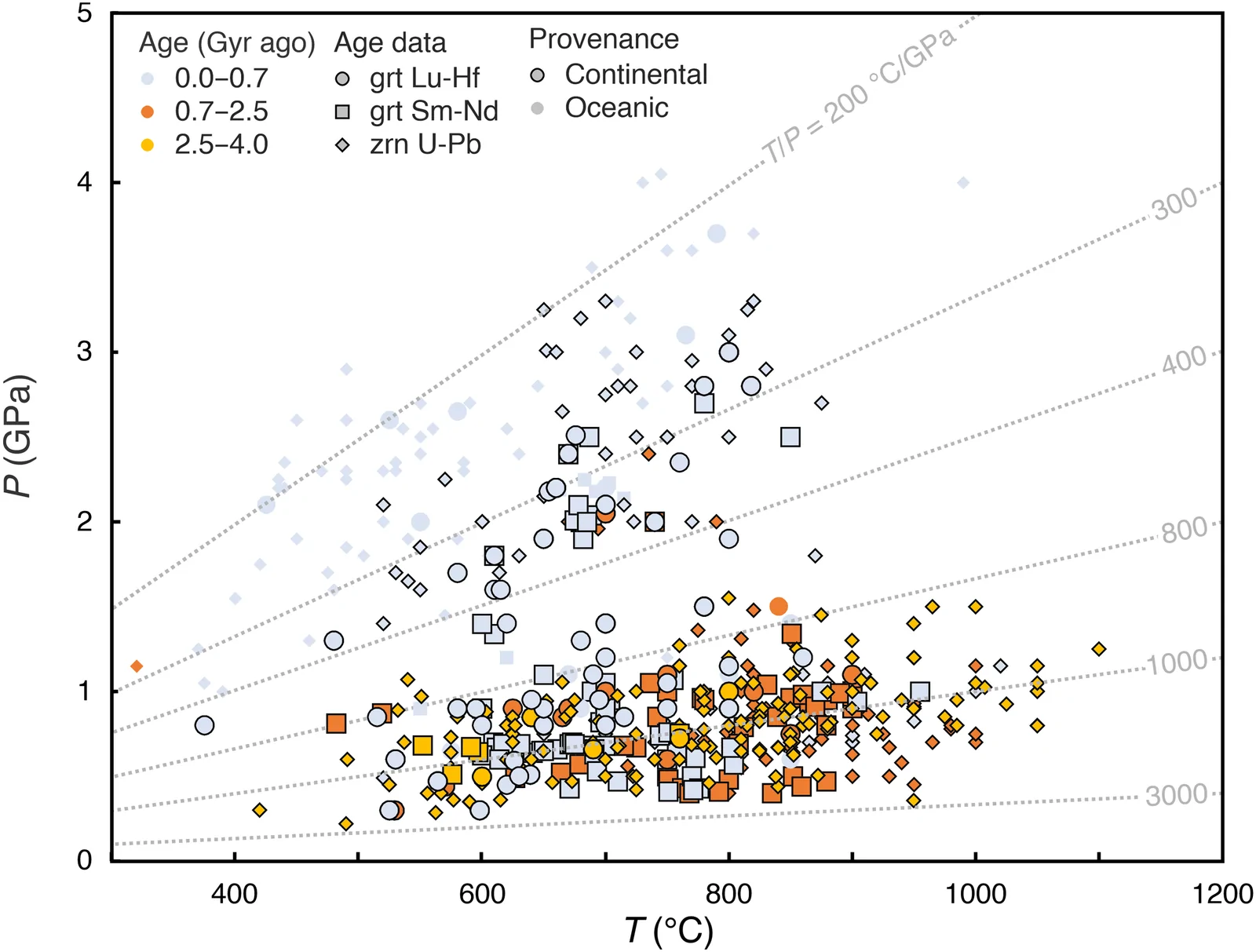
*P*-*T* estimates for metamorphic rocks. The data form the basis for the metamorphic record as established in Materials and Methods. Symbols with the outline represent continental and marginal rocks, whereas symbols without the outline represent oceanic rocks.

### Metamorphic mohometry

Orogenic crustal thickness and its expression in the metamorphic record are related to the tectonic process by which contractional orogenic systems develop. On the one hand, orogens can develop where the entire lithosphere is shortened and thickened and crustal thickening and horizontal shortening are directly linked (Fig. 3A). Such orogenic processes are related to far-field stresses acting at the base of the lithosphere and at plate boundaries and thus occur within plates (Tien Shan, Atlas), as well as in distal hinterland regions of active margins [Altiplano, Rocky Mountains, Tibetan Plateau; (*36*, *37*)]. The thickness of the ensuing crust depends on its strength and, in present-day settings, is regionally up to ∼60 km. These “distal” orogens differ from accretionary orogens, which develop within frontal regions of active margins, where plate-margin stresses directly drive shortening, and tectonic slices of up to several kilometers thick are scraped off the subducting lithosphere and added to a thickening orogenic wedge (Fig. 3B) (*37*). If the subducting lithosphere is oceanic, such wedges reach 30 to 40 km [e.g., Japan (*38*)]. If such systems progress to accommodate continental collision, the thickness of the accreted upper crust increases rapidly (e.g., Alps and Himalaya). Moreover, the lower-plate continental crust may horizontally underthrust the already thickened upper plate, leading to orogenic crust as thick as 85 km [e.g., Tibet (*39*)].

**Fig. 3.**
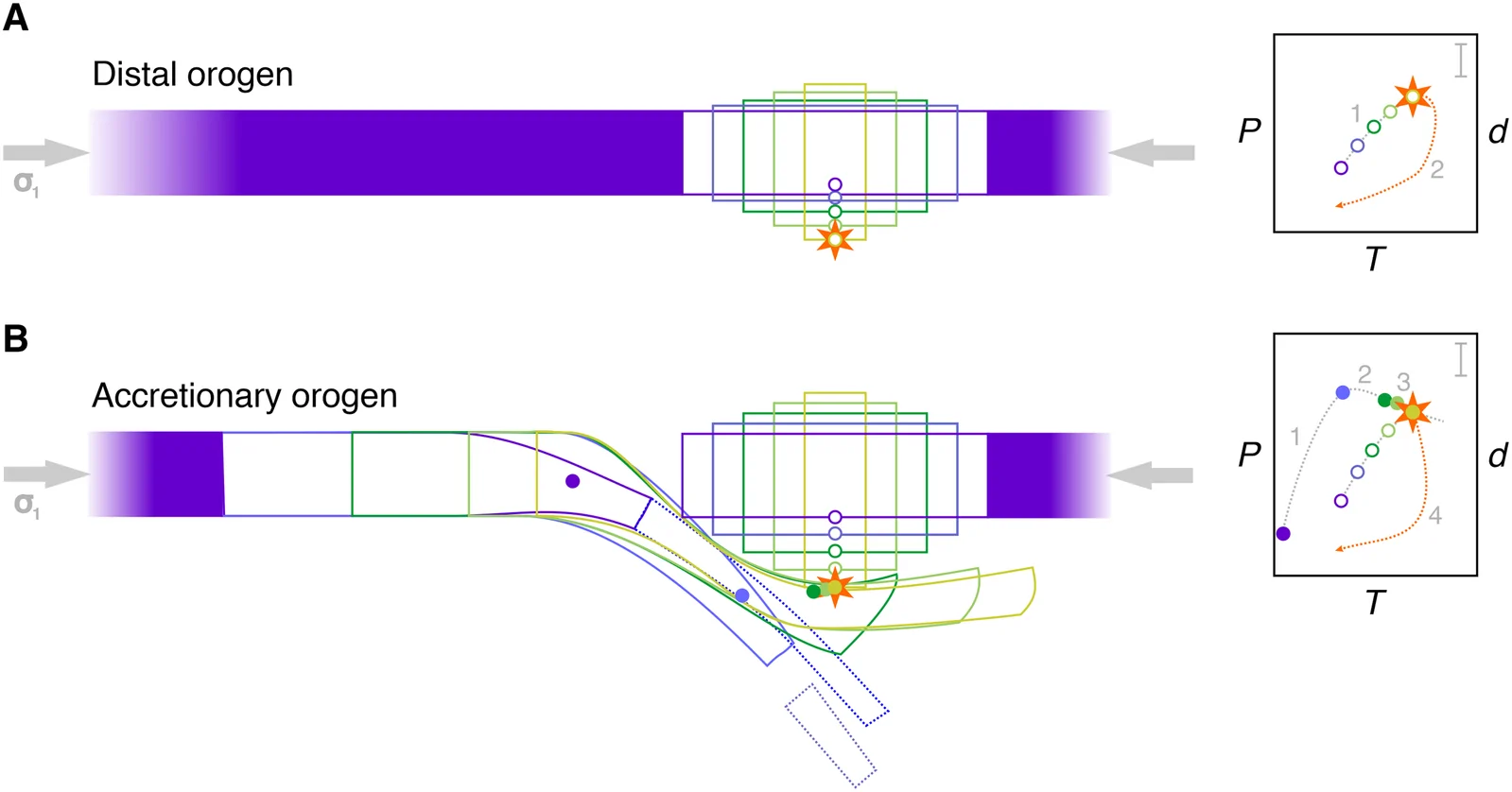
Schematic representation of metamorphic pressure, temperature, and depth in orogens. The diagrams show time evolutions of (**A**) distal orogens where shortening and thickening are directly linked and (**B**) accretionary orogens in the frontal region of active margins, which develop by subduction followed by continental collision and subduction. Markers track the evolution of the deepest crustal rocks that can ultimately be exhumed to the surface, with the orange star marking the deepest conditions recorded by these rocks. Equivalent depth *d* and typical uncertainties on *P* estimates are shown for reference. (A) Distal orogens thicken relatively homogeneously, with rocks exhumed from its deepest sections best capturing the thickness of the mature orogenic column. Stages in the *P*-*T* diagram are (1) thickening and (2) exhumation of the marker rock. (B) In accretionary orogens, the lower plate is buried into the mantle resulting from the slab from the still-attached oceanic lithosphere (1). Upon detachment from this lithosphere (2), the lower plate will bounce back and stall at the base of the developing accretionary orogen (3), where it will be pervasively overprinted and lose most if not all of the record of earlier, colder, and deeper burial. The marker indicates those parts of the ponded lower plate that will detach and exhume (4) as a large (U)HP terrane in the frontal part of the orogen.

The metamorphic record in the various types of orogens can be fundamentally different, the main difference being that in distal orogens, all rocks remain part of the orogenic pile, whereas in accretionary orogens, much of the lower-plate lithosphere may become subducted into the mantle. In the latter case, accreted rock slices of oceanic and continental margin provenance may be particularly deeply subducted and are either lost to the mantle or become exhumed as small (<1000 km^2^) ultrahigh-pressure (UHP) terranes that are buried quickly [<5 million years (Myr)] and undergo rapid, buoyancy-driven exhumation [3 to 6 cm year^−1^; e.g., UHP terranes in Ladakh, Western Alps, and Papua New Guinea (*40*)]. The continental lithosphere behaves fundamentally differently because of its strength and buoyancy. The continental crust can also be buried into the mantle. However, unlike with the oceanic or transitional lithosphere, deep continental subduction represents a transient state that only occurs during the first few million years of plate burial when continental margins may get dragged down into the mantle by a still-attached subducting oceanic slab (*41*). This process is reflected by low temperature/pressure (*T*/*P*; <250°C GPa^−1^) or UHP (>3.3 GPa) outliers in the *P*-*T* record of quintessential continental (U)HP terranes, such as the Western Gneiss Complex in Western Norway (*42*). Overwhelmingly more common in the metamorphic record of deeply buried continental terranes are high temperature (U)HP conditions (>700°C, *T*/*P* of 250° to 400°C GPa^−1^; Fig. 2) (*43*), which are imparted regionally when the buried crust bounces back up, stalls at the base of the orogenic wedge following slab break-off, and is heated up and pervasively overprinted (*42*). Upon stalling, parts of the continental crust may be thrust further beneath the orogenic hinterland (*44*), as occurred in Cenozoic time in the Zagros Mountains (*45*), Taiwan (*46*), Anatolia (*44*), and the Tibetan Plateau (*39*). Other parts detach and are exhumed relatively slowly over 15 to 30 Myr as large (U)HP complexes (>2000 km^2^) within foreland-directed thrust belts (*40*). Relics of this deep processing manifest as “hot eclogites”: mafic high temperature (U)HP rocks of continental provenance enclosed in felsic migmatites, granulites, and orthogneisses, which provide key archives for investigating the dynamics and depths of ancient orogenic root zones (*40*, *43*). Given the above, peak-*P* estimates from continental rocks provide an avenue for constraining Moho depth, because they best approximate the deepest levels of the orogenic crust (distal orogens) or the stalling depths beneath this crust with its Moho within barometric uncertainty (accretionary orogen). Such rocks are nevertheless most likely to be overprinted and least likely to be exhumed, which is why they are rare in the rock record. This is particularly the case in the Precambrian when orogens were hot and root zones were more prone to melt-assisted recrystallization and loss through delamination. Because of preservation bias and other caveats (Materials and Methods), the metamorphic record inherently is an incomplete series of imprecise and possibly biased snapshots of tectonic processes through time. Still, by extrapolating across time gaps and distinguishing consistency from anomaly, this record can be used to investigate the secular evolution of mountain belts.

## RESULTS

The metamorphic record established here includes existing and new thermobarometric and chronometric data obtained for global metamorphic rocks of continental provenance (Materials and Methods). For chronometric data, we rely on high-precision lutetium (Lu)-hafnium (Hf) and samarium (Sm)-neodymium (Nd) garnet ages where available, given that these may more unequivocally date metamorphism. Focusing on the maximum regional pressure, we observe that metamorphic rocks in Phanerozoic continental (U)HP terranes record consistent peak-*P* conditions of 2.8 to 3.3 GPa, exceeded only by local occurrences of rocks reflecting early transient passage into the mantle. These conditions indicate a common processing depth at the base of accretionary orogens, which—assuming lithostatic conditions—would be ∼90 km. This estimate matches closely the maximum crustal thickness estimated for the Pamir and Himalaya, which comprise the largest and thickest orogen that is still active [∼85 km; (*47*, *48*)]. Such *P* conditions are not recorded by continental rocks buried in the large, hot orogens of the Proterozoic. The few HP rocks that have been identified among these orogens instead record a relatively consistent maximum *P* of ∼2.0 GPa. These lower peak-*P* conditions indicate shallower maximum burial depths, which is consistent with lithospheric unzipping and shallow slab break-off preventing the development of thick orogens and the burial of continental crust into the mantle during this time (*44*, *49*). The maximum *P* conditions of ∼2.0 GPa would indicate a consistent maximum Moho depth of 55 to 60 km for these orogens (Figs. 1 and 2). This estimate is consistent with independent estimates of the maximum crustal thickness for the Grenville (1.2 to 0.9 Gyr ago), Picuris (1.47 to 1.37 Gyr ago), Yavapai (1.72 to 1.69 Gyr ago), and Trans-Hudson (2.1–1.8 Gyr ago) orogens, which were made using seismic restoration and chemical proxies (*50*–*53*). These results signify internal consistency among these various estimates and validate the geological interpretation of regional peak-*P* conditions for continental (U)HP terranes as reflecting basal stalling depths. Pressure conditions indicative of deep (>40 km) burial-exhumation cycles are not observed in the rock record before the Neoarchean (Figs. 1 and 2). Consistent peak-*P* estimates instead indicate maximum burial depths of ∼35 km in the Neoarchean and 30 km before this time. Thicker crust is nevertheless indicated by Pre-Neoarchean granitoids, which reflect melting of garnet- and rutile-bearing lower-crustal residues at depths of 40 to 45 km (*54*, *55*). This apparent discrepancy, which is also indicated by mass balance calculations, likely signifies preservation bias. The roots of the more mafic early crust comprised larger fractions of dense residual eclogite and thus were more prone to be lost to the mantle via delamination (*56*, *57*). This loss would have been exacerbated by the relatively high mantle potential temperatures and higher crustal densities in the Archean, which further favor the development of gravitational instabilities at the Moho (*58*), thus maintaining delamination as an effective means of crustal recycling (*55*, *57*).

Crustal thickness and elevation correlate with mean crustal density across geological settings and with crustal compositions and age (*7*). Using this empirical correlation, the crustal thicknesses that are indicated by *P* estimates can be recast into first-order estimates of paleo-elevation. Assuming an average density of 2.83 g cm^−3^ for modern andesitic continental crust (*59*), a maximum Moho depth of ∼85 km, as estimated for the modern Pamir and Himalaya on the basis of geophysical observations (*47*, *60*), indicates an elevation of 8.8_–0.7_^+0.7^ km above sea level, which is indistinguishable from the elevation of the three highest peaks (Mt. Everest, 8849 m; K2, 8661 m; Kangchenjunga, 8586 m). The same calculations yield a consistent elevation of 5.0_–0.6_^+0.6^ km for the large hot orogens of the Proterozoic. A theoretical elevation of 1.4_–0.5_^+0.4^ km would be obtained for the Neoarchean, whereas −0.1_−0.4_^+0.4^ km would be obtained for the Eoarchean, assuming a more mafic average Archean (proto)crust before 3.0 Gyr ago [average density of 2.87 g cm^−3^ (*61*); gradual transition to andesitic crust between 3.0 and 2.5 Gyr ago; (*62*, *63*)]. Whether these estimates are to be considered quantitatively depends on whether the mean sea level can reasonably be assumed to be constant. This assumption is valid for the Phanerozoic when the mean sea level likely did not differ by more than 300 m from that of present day (*64*). The same may apply for the Proterozoic, given that the factors that determine the mean sea level—ocean volume, continental volume and density relative to that of oceanic lithosphere, thickness of the oceanic crust, and relative height of spreading centers—are controlled by the geodynamic regime, which was kept at the steady state by plate tectonics. For the Archean, however, the mean sea level likely was vastly different, because (i) the crustal portion of the oceanic lithosphere was much thicker (*65*), (ii) the oceans were ∼1.5 times more voluminous because of seawater not yet having been recycled back into the mantle (*17*), and (iii) the Archean (proto)continental crust was more mafic, thus causing lower elevation through density inversion by widespread root eclogitization (*10*). It is thus remarkable or perhaps coincidental that the onset of crustal thickening capable of sustaining widespread emergence, as indicated by the metamorphic record, was broadly synchronous with emergence, as indicated by O (oxygen) isotopes in shales and strontium (Sr) isotopes in marine carbonates and stratiform barite deposits [3.5 to 3.0 Gyr ago; (*11*, *13*)].

## DISCUSSION

The evolution of metamorphic processes and conditions has long been recognized as broadly discontinuous, with two major revolutions ultimately driven by the secular cooling of the mantle (*15*, *49*, *65*). The first revolution (R1) occurred in the Meso- or Neoarchean when horizontal plate motion involving subduction and crustal thickening became the dominant tectonic style (*14*). Widespread “paired metamorphism”—with intermediate-*T*/*P* metamorphism (∼300°C GPa^−1^) in lower plates and high-*T*/*P* metamorphism (>600°C GPa^−1^) in upper plates of active margins—is a clear manifestation of this tectonic change (*14*), as are the global formation of passive margins (*66*), the assembly and reorganization of large continental landmasses (*19*, *20*), and the production of large, long-lived orogens formed by accretion and intraplate thickening (*20*). The second revolution (R2) occurred toward the end of the Proterozoic when steady cooling of the mantle resulted in a myriad of geodynamic changes, including (i) deeper slab break-off and increased slab pull; (ii) a thicker oceanic lithosphere capable of coherent, long-term subduction; and (iii) a departure from default lithospheric unzipping in orogens, thus leaving in place the downward force needed to subduct the buoyant crust into the mantle in accretionary orogens (*44*, *49*). These changes marked the terminus of the “hot orogens” of the Proterozoic, with their characteristic ultrahigh temperature metamorphism and massif-type anorthosites, and led to the establishment of the modern geodynamic regime, expressed in the metamorphic record as the emergence of continental (U)HP rocks and low-*T*/*P* (<300°C GPa^−1^) oceanic blueschists and eclogites (*14*–*16*, *49*). The deep underthrusting of continental margins that has been prevalent since R2 invited the formation of large accretionary orogens, which represent the thickest orogens ever made. Evaluation of the metamorphic record indicates that R2 represented a distinct period of orogenic growth after which the crust in orogenic belts was able to become up to twice as thick and support ∼4 km of additional elevation. This change marked a shift from orogens with intermediate-altitude (∼4 km) terrain to high-altitude mountain ranges akin to the present-day Pamir-Himalaya-Tibet orogen. Orogenic growth associated with R1 is much less well resolved in time and space and may have been much more protracted. This long period of growth nevertheless also appears to have allowed the orogenic crust to become about twice as thick as before, likely sustaining Archean emergence (*11*–*13*) and the formation of the first mountainous regions in the Paleoproterozoic (*52*).

Causes for environmental upheaval culminating in the GOE and NOE have been sought in various external forcing mechanisms, such as increased runoff resulting from supercontinent breakup, enhanced weathering of basaltic rocks at low latitude, and a dwindling CO_2_ (carbon dioxide) flux from arc volcanism (see Supplementary Text). Although each is a valid hypothesis, these processes are largely transient, which does not match the irreversible nature of global oxygenation and other key changes to the Earth system state. The role of these processes as external forcing on the environment can be further called into question, because they are not restricted to the periods leading up to the GOE and NOE; similar processes have occurred throughout Earth’s history seeming without any major environmental consequence. The apparent absence of time-consistent external-forcing mechanism in the geologic record inspired the development of models that can explain global oxygenation through internal biochemical feedbacks (*50*). In contrast to all of these processes, the orogenic growth associated with tectonic revolutions R1 and R2 broadly coincide with the major periods of environmental upheaval in terms of their approximate timing, paucity, and irreversibility (Fig. 1). Hypotheses that may explain this consistency can be readily posed. Orogenic growth would have raised global terrigenous runoff and nutrient fluxes. This effect would be compounded for the first growth period by continental emergence and crustal growth, both of which would have raised continental weatherability. Seawater Sr records likely track these flux increments (*21*), showing marked increases in the proportion of terrigenous Sr relative to the total Sr delivered to the oceans during the orogenic growth periods (Fig. 1). Similar changes would apply to phosphorus (P), which is similarly incompatible and enriched in the upper crust as Sr and is likewise sequestered in relatively weatherable carbonate and phosphate minerals. The enhanced terrigenous element flux would have raised primary productivity, given that the P supply is rate-limiting and terrigenous runoff contains many other bioactive (micro)nutrients such as Ba, Zn, Fe, and Si. This effect would be amplified by an increase in the SO_4_^2−^ supply available for the release of bioactive P from remineralizing marine biomass (*67*). Consequences of an enhanced flux of P would have been particularly impactful in Archean surface waters, where P concentrations were low as a result of anoxic and ferrigenious conditions favoring Fe-P trapping and P exhaustion through photoferrotrophy (*23*, *67*). These flux increments could explain the major biological innovation recorded by the S isotope record of marine pyrite, which reflects increases in marine S metabolism during R1 and R2, each later followed by an increase in global biomass and biodiversity (Fig. 1) (*33*). Other effects of enhanced runoff would have been (i) oxygenation from increased burial of organic carbon (*68*), (ii) an increased CO_2_ flux to the atmosphere from accelerated oxidative weathering of rock-bound organic carbon (*69*), and (iii) increased pH and salinity of ocean waters from an enhanced terrigenous supply of alkali metals. These considerations motivate posing the overarching hypothesis that orogenic growth during R1 and R2 served as a key endogenic driver for the development of a habitable environment during the past 3 Gyr.

Apparent temporal inconsistencies between the periods of orogenic growth and various proxy changes [e.g., *V*_bio_ and atmospheric partial O_2_ pressure (*P*o_2_); Fig. 1] exist. These inconsistencies could be taken as disproving the hypothesis, but they could also signify that Earth system responses to tectonic change were complex and multifaceted, and relied on delicate shifts in limiting (bio)chemical feedbacks. Instead of keeping pace with increases in nutrient flux, different components of this response likely showed threshold behavior or otherwise nonlinear progression (e.g., *P*o_2_ and *V*_bio_). The overall response likely would have differed between growth periods as well, given the geodynamic and Earth system changes that occurred in the interim. R2, for instance, occurred in a largely oxygenated world with large emerged landmasses, which was profoundly different from the situation in which R1 unfolded. This complexity is set against the limited data resolution of the Precambrian metamorphic record, which impedes precise constraints on the timing and tempo of orogenic growth during R1 and R2. The resolution is particularly limited for the first growth period, which is only broadly constrained in time and may comprise several individual growth spurts spaced hundreds of millions of years apart rather than a single period of continuous change. Thus, while the observations made here are consistent with the hypothesis of tectonic forcing, the robustness of this hypothesis needs to be tested. Avenues for doing so may lie in the following: (i) a more complete global database of metamorphic pressure data with focus on Precambrian terranes, (ii) refined and expanded (bio)chemical proxy records targeting the broader R1 and R2 time windows, (iii) statistical testing for temporal relationships between tectonic change and biogeochemical signals, and (iv) quantitative Earth system modeling to investigate forcing effects of nonlinear increases in terrigenous runoff and P fluxes through time.

## MATERIALS AND METHODS

### Building the metamorphic record (1): Approach and caveats

Establishing the metamorphic record requires accurate constraints on the timing of metamorphism. So far, this has mostly been done using age data from zircon, which is the most widely used chronometric technique. Although zircon provides by far the richest resource of age constraints, there are potential limitations because of possible lithological bias, age misattribution in cases of polymetamorphism, and the general difficulty of linking zircon (re)crystallization to *P*-*T* conditions. Garnet provides a useful alternative resource in this regard. Like with zircon, precise and accurate age data can be obtained from garnet using chronometers that are relatively robust against thermal reequilibration [^176^Lu-^176^Hf and ^147^Sm-^143^Nd (*70*–*72*)]. Unlike with zircon, garnet is typically metamorphic and directly provides *P*-*T* estimates, limiting the risk of misattributing ages to metamorphic events (*73*–*77*). Using garnet-based data for time series analysis of the metamorphic record thus provides additional constraints, as well as an independent check for possible age misattribution. Bulk-grain Lu-Hf and Sm-Nd geochronology enables high-precision age constraints (typically <0.5% 2SD), which permit extreme age resolution for time series analysis. These data were used in conjunction with robust peak *P*-*T* estimates obtained from the same sample or similar samples from the same tectonic terrane or unit. At a first order, the garnet-based record is broadly similar and complementary to that obtained using uranium (U)-lead (Pb) zircon ages (Fig. 1). Where both zircon U-Pb and high-precision garnet Lu-Hf or Sm-Nd are available, both broadly date the same orogenic stage, with some notable exceptions for several Proterozoic HP rocks, where metamorphism was misattributed to the Archean on the basis of zircon. These and other details of the garnet-based metamorphic record are discussed below.

Consideration was given whether to expand the garnet-based record with results obtained using new methods of in situ Lu-Hf and U-Pb garnet dating (*78*, *79*). Although growing fast, these techniques are still in their infancy, and the amount of data from these techniques is still small at this stage. Additional factors to consider are that in situ age results are inherently less precise (typically ≥3% 2SD) and are typically obtained from subsets of data selected on the basis of interpretive or statistical arguments. Causes for dispersion and general accuracy are difficult to verify given the still-limited number of available garnet reference materials for either method. For the analysis done in this study, we deem the advantages of unilaterally including in situ garnet age data—slightly increased number of data and coverage—to not yet outweigh the uncertainty associated with some of the results. Instead, we highlight specific results obtained recently for Archean terranes where high-precision garnet age data are not yet available. With rapid development and applications of in situ techniques, it is expected that testing and refinement of garnet-based time series analysis using in situ data will be valuable in the future.

Using garnet- and zircon-based metamorphic data, a basic approach to building a time-resolved record of metamorphic processes and possible burial depths would be to survey the maximum *P* conditions and to take these as proxies for burial depth. This approach faces several inherent limitations and caveats that are intrinsic to the metamorphic record (*49*): (i) Metamorphism is punctuated, with recrystallization processes typically occurring only when active tectonic processes are still ongoing. (ii) The metamorphic record is inherently discontinuous. Active mountain building and associated metamorphism are not ubiquitous. Furthermore, selection bias in exhumation processes; preservation bias due to selective overprinting, especially of deep rocks; and sampling bias may cause signals from specific orogens to be obscured, overlooked, or even entirely erased. These biases particularly affect old rocks, which are most likely to be overprinted, and rocks from the basal levels of mountain belts where the chances of material making it back to the surface are particularly low. (iii) Rayleigh-Taylor instabilities at the base of the orogenic crust may cause rocks to transiently reside at depths that exceed the regional Moho depth (*80*, *81*). (iv) *P*-*T* estimates may be problematic. *P*-*T* estimates may be much less reliable than indicated by analytical uncertainties alone and may be in error when based on questionable mineral modes and activity estimates or the composition of minerals that may not have reached equilibrium. (v) Records of active orogens may be biased, because the deepest domains of the orogenic crust may not (yet) be entirely exhumed and exposed (e.g., Himalaya-Tibet orogen). Crustal xenoliths in magmatic rocks may be used instead to gain insight into the still-buried parts of orogens, but these are rare, even in modern orogenic settings, and their *P*-*T* and petrological record may be affected by melt-rock interaction.

A more fundamental issue pertains to the use of *P* estimates as a proxy for depth. In general, lithostatic pressure is assumed where *P* equals the product of the thickness and average density of the overburden and the gravitational acceleration. This generally amounts to 0.3 GPa km^−1^ for most of the crust and uppermost mantle, which is widely used to convert *P* to burial depth. This assumption, however, is likely not to hold in many parts of the lithosphere. Contributions from deviatoric stresses to the overall stress state of rocks may cause large differences between the apparent depths that would be estimated from thermobarometric *P* estimates assuming lithostatic conditions and the actual depth where the rock resided (*82*–*84*). Being case-specific, such differences are expected to manifest as scatter in the *P* record of metamorphic terranes and the metamorphic record in general.

Given the various limitations, the metamorphic record, as obtained from continental rocks exhumed or erupted from the depths of mountain belts, is to be approached conservatively as an incomplete series of imprecise snapshots of the long-term evolution of orogens and orogenic processes, with particular underrepresentation of old and deep rocks. Examination of this record in the global context is a matter of extrapolating across time gaps and distinguishing consistency from anomaly to determine the general trends that are geologically meaningful and relevant. Acknowledging the above, we developed a time-resolved record of metamorphism using garnet-based data (table S1) complemented with zircon-based data from existing compilations (*85*, *86*), with the addition of new *P*-*T* and age data obtained recently for Rhyacian HP rocks in Cameroon (*87*) and the Democratic Republic of the Congo (*88*). The literature compilations were screened and filtered only for (i) data that represented the mantle rather than crustal rocks and (ii) data based on *P*-*T* conditions that could not be verified in publications. Further details regarding this record and the characteristics for each eon are provided below.

### Building the metamorphic record (2): Time series analysis

#### Archean

Constraints for the Archean are few yet they are crucial for the characterization of early metamorphism. The earliest examples of this are therefore discussed individually and in detail, followed by a more general survey. The age record from metamorphic garnet starts in the Eoarchean with the results obtained for the Isua Supracrustal Belt (ISB) and Itsaq Gneiss Complex (IGC) in West Greenland. The earliest results for the ISB [Sm-Nd ages of 3.72 ± 0.02 Gyr ago; (*89*)], however, have garnered scrutiny, given their relatively complex nature. New Lu-Hf results from the ISB cast doubt on the interpretability of these results and instead indicate a Neoarchean age of metamorphism and shearing of the ISB [2.6 to 2.4 Gyr ago; (*90*)]. Eoarchean garnet ages were otherwise obtained from amphibolites (peak *P*-*T* conditions: ∼0.9 GPa and 780°C) from the adjacent IGC (*91*). The in situ Lu-Hf ages (3.65 to 3.58 Myr ago) are uncertain and may reflect partial disturbance. They nevertheless provide a strong indication that the low-*P*, high-*T*/*P* (840° to 950°C GPa^−1^) metamorphism, which affected the IGC and is common for much of the Archean, was of Eoarchean age. The second oldest known occurrence of garnet-grade metamorphism is in the Acasta Gneiss Complex (AGC; Northwest Territories, Canada). The AGC is widely recognized as a polymetamorphic terrane with a range of Archean metamorphic imprints recorded from 3.75 Gyr ago onward, as well as Paleoproterozoic overprinting relating to the Wopmay Orogeny. In situ Lu-Hf analysis of garnet from tonalite gneisses within a low-strain enclave of the AGC provided ages between 3.28 and 3.21 Gyr ago, as well as 1.86 ± 0.04 Gyr ago (*92*), showing that the terrane was metamorphosed (∼750°C and 0.5 GPa) during the Paleoarchean and was reworked in the Paleoproterozoic and possibly in between. The Paleoarchean age resembles the high-precision Lu-Hf age obtained for garnet in the Stolzburg terrane of the Barberton Granite-Greenstone Belt (3.23 ± 0.02 Gyr ago), which reflects metamorphism at a slightly higher *P* [∼640°C and 0.9 GPa; (*93*)]. The dispersion in the AGC in situ data could reflect the ∼1.86-Gyr-ago overprint but could also signify additional Archean reworking.

The search for early high-*P* metamorphism in the rock record has been ongoing for a long time. Eclogitic rocks of oceanic and continental provenance in the Belomorian Province (Kola Peninsula, Russian Federation) have yielded U-Pb zircon ages of ∼2.87 Gyr ago and have hence been argued to represent a very early occurrence of high-*P* metamorphism (*94*). One of these occurrences (“Gridino-type eclogite”) is argued to derive from mafic dykes of continental provenance, thus suggesting Archean deep continental subduction to peak conditions of ∼700°C and >1.3 GPa. Garnet Lu-Hf dating of the oceanic (“Salma-type eclogite”) and continental eclogites, however, consistently yielded Paleoproterozoic ages 1901 to 1878 Myr ago (*73*, *93*), as did further U-Pb dating and rare earth element analysis of zircon [1.91 to 1.88 Gyr ago; (*73*)(*95*)] and recent in situ Lu-Hf garnet dating [1.88 ± 0.09 Gyr ago; (*96*)], indicating a case of age misattribution by Mints *et al.* (*94*). A similar case of age misattribution occurred with garnet clinopyroxenites in the Zunhua ophiolite mélange. These rocks of oceanic provenance were interpreted as retro-eclogite with peak metamorphism in the Neoarchean (∼2.47 Gyr ago) constrained by U-Pb zircon dating (*97*). Reevaluation and garnet Lu-Hf and Sm-Nd dating of these rocks showed them to be amphibolite-facies rocks (peak *P*-*T* of ∼0.5 GPa and 550°C) metamorphosed at 2.10 Gyr ago, with older zircon ages representing relics from the protolith (*77*).

A case for Archean high-*P* metamorphism may be provided by metasedimentary rocks from the Lewisian Gneiss Complex (LGC), northwest Scotland. The metamorphic assemblages in these rocks reflect *T*/*P* < 440°C GPa^−1^, which is lower than the values reflected by most Archean metamorphic rocks (>675°C GPa^−1^). In contrast to the Belomorian eclogites, Lu-Hf garnet dating indicated a Neoarchean age (2.81 ± 0.04 Gyr ago) for the metamorphism of these rocks (*98*). Uncertainty in this case, however, stems from the *P*-*T* estimates and petrological interpretation of the samples. The *P*-*T* estimates that were obtained for the analyzed samples (580° to 660°C and 1.5 to 2.5 GPa) are highly uncertain and different from those constrained so far for Lewisian crustal rocks, all of which indicate peak-*P* conditions below 1.5 GPa (*99*–*102*). Evaluating the cause of this difference at this point is difficult. On the one hand, the rocks may represent an occurrence of HP metamorphism. On the other hand, the HP conditions may be questioned, because (i) they are based on empirical phengite barometry, which is inherently uncertain; the Si contents seen in LGC white mica [3.2 to 3.5 a.p.f.u. (atoms per formula unit)] are more prevalent under HP conditions but also occur below 1.5 GPa (*103*). In addition, (ii) omphacite, which thermodynamic modeling predicts to occur in the rocks, was not observed. Because of the above and the large uncertainty in *P* estimates, the LGC datum at this stage does not provide the necessary resolution for time series analysis and was hence not included in Fig. 1B. Other than the above, the metamorphism recorded before the Neoarchean is typified by high *T*/*P* (>675°C GPa^−1^) and peak-*P* conditions at or below 1.0 GPa, with a few exceptions from the Pilbara and Barberton granite-greenstone belts, which reached 1.1 to 1.2 GPa in the Mesoarchean. This changed going into the Neoarchean when occurrences of metamorphism at ∼1.0 GPa became more common and several continental terranes reworked in large tectonic zones record *P* conditions of up to ∼1.5 GPa.

#### Proterozoic and Phanerozoic

A new steady state in the peak-*P* conditions of metamorphosed continental crust appears to have been reached in the Paleoproterozoic, with the occurrence of high-grade metamorphism of the Nyong Complex in South Cameroon [∼2.09 Gyr ago; (*87*)], the Kasai-Lomami Supergroup in the South of the Democratic Republic of the Congo [∼2.06 Gyr ago; (*88*)], Usagaran Belt in Central Tanzania [∼1.99 Gyr ago; (*104*)], the former eclogites of the Belomorian Province (∼1.88 Gyr ago; see above), and the Ungava Orogen in North Québec, Canada [∼1.83 Gyr ago; (*105*)]. Garnet ages are available for at least two cases (Usagaran and Belomorian HP rock). Most of these rocks reflect peak-*P* conditions of ∼2.0 GPa, except the mafic rocks from the Ungava Orogen, for which an estimate of 2.5 ± 0.2 GPa has been proposed. As burial of crust into the mantle is unlikely during this time (see the main text), such relatively high *P* could instead be taken to indicate relatively extensive crustal thickening or transient overthickening or could represent a local pressure anomaly (*17*). Alternatively, the estimate itself may be less certain than indicated. The mafic rocks in question are reported to contain relict omphacite, and much of the matrix is argued to have comprised this HP phase (*105*). Nevertheless, because of the strong retrogression, a peak assemblage could not be fully established. The *P* of ∼2.5 GPa was estimated on the basis of garnet modal abundance, as determined by point counting on a section of rock. There are two potential issues with this: (i) This approach could yield biased results given the coarse grain size and heterogeneous distribution of garnet in the rock, and (ii) scrutiny of the point-counting results shows that many domains were counted as garnet, even though they are not and may not have been. Possible *P* overestimation would be consistent with the jadeite component of omphacite, which is too low for this rock at 2.5 GPa and instead indicates ∼2.0 GPa. We thus conclude at this point that Paleoproterozoic HP rocks reflect a relatively common peak *P* of ∼2.0 GPa and that the estimates obtained so far for the retro-eclogites of the Ungava Orogen are not constrained well enough to disprove this hypothesis. In the absence of indications otherwise, the increased peak-*P* conditions recorded by the Paleoproterozoic HP rocks may be considered the product of a steady increase that started in the Neoarchean with the first emergence of rocks buried to 1.1 to 1.2 GPa. It cannot be excluded that this change, which occurred over a very long period of time (>0.5 Gyr), was stepwise, rather than continuous, and involved several pulses of metamorphic change. The end-result of this change is nevertheless clear and sets Paleoproterozoic metamorphism apart from that seen before the Neoarchean.

The metamorphic record of the Mesoproterozoic is dominated by rocks processed in a network of large hot orogens that developed as part of Rodinia assembly, most prominently the Grenville-Sveconorwegian orogen. As with Proterozoic metamorphic rocks in general, the vast majority of Mesoproterozoic rocks reflect the low-*P*, high-*T*/*P* (≤1 GPa; >675°C GPa^−1^) metamorphism that typifies this larger orogenic system. Much of the Grenville-Sveconorwegian appears to have been too hot and ductile to thicken substantially beyond the thickness indicated by these pressures. Nevertheless, several occurrences of HP rocks of continental origin occur in this broader orogenic system, all of which show peak-*P* conditions similar to those recorded by the Paleoproterozoic HP rocks described above [∼2.0 GPa; (*106*–*109*)]. The eclogitic rocks that record these conditions underwent relatively high temperatures (740° to 870°C) and are typically migmatitic or restitic. Exhumation of these rocks can often be ascribed to ductile extrusion, by which a part of the hot and partially molten and eclogitized lower crust was transported toward and through the orogenic wedge, exhuming as a large, intensely deformed nappe (*110*). Most of these rocks exhibit a pervasive granulite-facies overprint associated with the latter stage of their history, clearly reflecting the relatively extreme thermal state that typifies the large hot orogens of the Proterozoic.

It cannot, at this stage, be quantitatively assessed whether and to what extent these high-*P* rocks are representative of the orogen where they were found or the Grenville Orogen in general. Preservation and sampling bias invalidate using the quantity of a certain type of metamorphic rock to argue for the prevalence of a specific orogenic process or setting. This especially applies to high-*P* rocks in this case, as the rocks within root zones of hot orogens are likely particularly susceptible to loss through delamination and to overprinting given the widespread presence of melt and intense deformation associated with melt-assisted flow at high temperature. Uniform treatment requires us to consider the HP rocks of the Grenville as valid proxies of how thick parts of the Grenville Orogen were able to become before collapsing, bearing in mind that the prevalence of thickened domains may have been lower than they have been in younger orogens and, thus, that the global orogenic change in the Neoproterozoic—in terms of the sheer increase in area occupied by tall mountains—may have been more profound than the *P* growth curve suggests. High-*P* continental rocks recording similar 2.0-GPa peak-*P* conditions as Grenville(-affiliated) HP rocks occurred well into the Neoproterozoic—notably in the Egéré-Aleksod Terrane in southern Algeria [∼685 Myr ago; (*111*, *112*)] and the Richarddalen Complex in northwest Spitsbergen [∼620 Myr ago; (*113*–*115*)]—but peak-*T* conditions reflected by these rocks are generally lower (<700°C).

The first sign of a major change in orogenic style is provided by the eclogites of the Ceará Group in the Brasiliano Orogen, northeast Brazil [∼605 Myr ago; (*116*–*118*)]. These rocks underwent peak-*P* conditions of ∼2.8 GPa, which is far higher than any *P* recorded until then by continental rocks. The rocks appear to usher in the modern age of continental (U)HP metamorphism. Continental terranes that have since become part of the lower plates of accretionary orogens reflect similar or even higher pressures. Although single eclogite or diamond-bearing gneiss occurrences among these terranes may indicate much higher *P* conditions (>3.7 GPa), most eclogites in these terranes reflect a common 2.8- to 3.3-GPa imprint. These prevalent *P* conditions can be ascribed to the widespread reworking of these terranes at the base of orogenic wedge (*21*) and are observed in all continental UHP terranes of the Phanerozoic, including those in the Northern Qaidam Orogen, northern China [∼435 Myr ago; (*119*, *120*)]; the Western Gneiss Region, western Norway [∼410 Myr ago; (*121*, *122*)]; the Sulu Belt, southeast China [∼230 Myr ago; (*123*, *124*)]; and the youngest-known UHP rocks exposed on the d’Entrecasteaux Islands, eastern Papua New Guinea [∼7 Myr ago; (*125*, *126*)]. While not yet exhumed from the Pamir-Himalaya-Tibet orogen, continental rocks of predominantly felsic composition reside at depths consistent with such pressures within the orogenic root zone (*127*).

## Supplementary Materials

### The PDF file includes:

Supplementary Text Legend for table S1 References
