# Supplementary material for: Raising mountains for a habitable Earth
Source: Sci Adv. 2026 Nov 13;12(46):eaei2764. doi: 10.1126/sciadv.aei2764 (PMC13763938; doi:10.1126/sciadv.aei2764)
Supplement: Supplementary file 1 [file sciadv.aei2764_sm.pdf]

Science Advances
AAAS

Supplementary Materials for

Raising mountains for a habitable Earth

Matthijs A. Smit *et al.*

Corresponding author: Matthijs A. Smit, msmit@eoas.ubc.ca

Sci. Adv. **12**, eaei2764 (2026)
DOI: 10.1126/sciadv.aei2764

The PDF file includes:

Supplementary Text
Legend for table S1
References

Other Supplementary Material for this manuscript includes the following:

Table S1

Supplementary Text – Earth System context for Figure 1

The main changes to the Earth system state are summarized in Figure 1 of the main text. Below, context is provided to these changes to further illustrate the intricate interconnection between these changes. The rise of oxygen levels in the global oceans and atmosphere as shown in Figure 1 was complex stepwise process, with many intervals of local and global change. Of these various events, the two most substantial and impactful changes occurred in the wake of the Archean and in the Proterozoic. From c. 3.0 billion years ago onward, oceanic oxygen levels started to rise. This pivotal change is recorded in various natural archives, including biomarkers, sedimentary records, and the isotope compositions and elemental concentrations of redox-sensitive elements, such as S, Cr, Mo, Re, and U; *24*, *27*, *29*, *128-132*). Steady oxygenation during the Meso- and Neoarchean culminated in the Great Oxidation Event (GOE; 2.4-2.2 Gyr ago; *28*), when atmospheric oxygen concentrations (pO_2) rose rapidly, and the mass-independent S isotope fractionation ($\Delta^{33}S$) and Xe isotope fractionation signatures of global anoxia disappeared indefinitely (*25*, *133*). Global oxygenation during the Neoarchean to Paleoproterozoic was fundamentally driven by the start of oxygenic photosynthesis, with facilitation by many other factors. The GOE may have been propelled by increased submarine volcanism and changing composition of volcanic gases, the escape of H_2 to space, shifts in the composition of the exposed crust (*63*, *134-138*). The second stage of global oxygenation (NOE) has been identified through distinct changes in the total organic content and the concentration of redox-sensitive elements (e.g., V, Mo and U) of black shales, and the S isotope compositions and of marine sulfides and sulfates; *27*). The NOE has been ascribed to changes in global plate configuration and the development and diversification of algae and other micro-organisms (*27*, *28*).

The reverberation of the global oxidative events across the terrestrial environment and marine water column is reflected in the S isotope record, which is coupled to the O cycle via various chemical and biochemical processes involving sulfate-sulfide reactions (*139*). Global atmospheric oxygenation during the GOE is recorded in the loss of the signatures of mass independent S isotope fractionation (non-zero $\Delta^{33}S$) from the rock record (*25*). This relatively abrupt change reflects the threshold behavior associated with the shift in the S cycle from being strongly influenced by atmospheric gas-phase reactions to being governed by oxidative weathering. The $\delta^{34}S$ values (permille deviation in $^{34}S/^{32}S$ relative to a standard) of marine pyrite provide a more nuanced view on the S cycles and S metabolism in association with the emerging oxygenation of the oceans before the GOE (3.0-2.5 Gyr ago; *129-132*). Changes in the $\delta^{34}S$ values of marine pyrite may in part reflect differences in sedimentation rate, the concentration of dissolved C, S and Fe, and the degree of Fe and S remobilization within the sediment that hosts the pyrite (*139*). However, an overwhelming effect on pyrite $\delta^{34}S$ values is caused by biological S metabolism, which can cause positive or negative isotope excursions depending on the exact microbial pathway and process (*140*). The $\delta^{34}S$ values show an increasingly larger range ($\Delta^{34}S$; permille difference between $\delta^{34}S_{max}$ and $\delta^{34}S_{min}$ for samples from a given time interval) with significantly more negative and positive values from c. 3.0 billion years ago onward (determined on the basis of a global compilation of c. 4,450 S isotope data; *22*). The range in $\delta^{34}S$ values ($\Delta^{34}S$) shows two distinct increases, the first occurring over a protracted period from c. 3.0 billion years ago culminating in the GOE and the second broadly during the NOE (Fig. 1A). Significant increases in global biomass and biodiversity occurred after both stages, showing both the limiting role of O_2 on the evolution of life (*33*) and the complex threshold behavior of the biological response to environmental change.

Beyond impacting S and biochemical cycles, enhanced oxidative weathering in the wake of the GOE drove the burial of large amounts of organic carbon (*28*, *141*). Drastic positive excursions in $\delta^{14}C$ recorded in global carbonate deposits formed between 2.31 and 2.06 billion years ago (the Lomagundi-Jatuli Excursion) reflect this carbon, which is characterized by low $\delta^{14}C$ due to photosynthetic fixation (*26*, *142*, *143*). Rapid carbon burial during this time was likely aided by an increasing abundance of shallow basins (*144*) following the emergence of the continents c. 3.0 Gyr ago (*11*, *13*) and a flood of bio-limiting nutrients to these basins and the global ocean (*28*, *29*). Further impacting greenhouse gas levels, rising O_2 levels drove atmospheric CH_4 dissociation and suppressed methanogenesis of organic matter by microbes—the latter adding to the curtailing of biogenic methanogenesis by drastically declining the supply of bioactive Ni to the oceans (*145*). These processes, together with a CO_2 drawdown driven by enhanced weathering of flood basalts formed at low latitude (*29*), plunged the world into a global icehouse (Fig. 1A), with widespread glaciation occurring episodically in between pulses of rising pO_2 (Huronian Glaciation; *28*, *29*, *31*, *134*). A similar scenario unfolded in the wake of the NOE (Fig. 1A), when enhanced continental runoff due to supercontinent breakup and flood basalt eruption at low latitude, and a dwindling CO_2 flux from global arc volcanism led to a catastrophic CO_2 drawdown that ultimately triggered two distinct "Snowball Earth" glaciations – the Sturtian (717-659 Myr ago) and Marinoan (650-635 Myr ago) Glaciations (Fig. 1A; *29*, *30*, *32*, *146*). Barring the clear difference of no pre-existing methane greenhouse in the Neoproterozoic, similarities between the GOE and NOE could indicate that both changes share climatic feedback mechanisms (*29*). Although yet to be fully characterized, it is likely that these mechanisms that are the reason why the two most severe icehouse episodes followed the two most substantial oxidative events in Earth's history.

The drastic changes summarized above are set against the backdrop of the evolving solid Earth, with its cooling mantle, differentiating crust, and evolving geodynamic regime and plate configuration (*9*, *14*, *15*). The proto-continental crust of the Eoarchean was relatively mafic (Fig. 1B), still retained ^{142}Nd and ^{182}W excesses from early differentiation, and mostly developed through the volcanic resurfacing of high-U/Pb basalts, internal differentiation, and delamination (*12*). This changed from 3.2 billion years ago onward, with the widespread development of juvenile crust of largely tonalite-trondhjemite-granodiorite (TTG) composition (*147*) and the steady development of a more andesitic upper crust (Fig. 1B; *62*, *63*). This change likely links to a shift from a primitive single-lid regime with temporal "squishy-lid" or mobile-lid geodynamics to a mode more akin to modern-day plate tectonics (*12*, *148-155*). Although Archaean TTGs likely reflect melting of crustal roots, rather than actual arc magmatism (*35*, *156-158*), the hallmarks of subduction emerged in the geological record of this time (*155*, *159*, *160*). Global plate tectonics set in motion the supercontinent cycle, which involved the assembly and breakup of pan-continental landmasses between 2.7-2.4 billion years ago (Superia and Vaalbara, Ur, or Kenorland), 2.1-1.8 billion years ago (Columbia or proto-Nuna), 1.6-1.4 billion years ago (Nuna proper), 1.1-0.8 billion years ago (Rodinia), 0.6-0.4 billion years ago (Gondwana), and 0.3-0.2 billion years ago (Pangaea; *18*, *19*, *161-163*). Various links have been proposed between the evolving solid Earth and the Earth system state. Possible external-forcing mechanisms in this regard may be increased runoff due to supercontinent breakup, enhanced weathering of basaltic rocks at low latitude, and a dwindling CO_2 flux from arc volcanism (*29*, *30*, *129*, *130*, *136*, *140*, *164-166*).

Table S1: *P-T* and age data used in this study (provided separately as .xlsx).

REFERENCES

1. N. J. Planavsky, O. J. Rouxel, A. Bekker, S. V. Lalonde, K. O. Konhauser, C. T. Reinhard, T. W. Lyons, The evolution of the marine phosphate reservoir. *Nature* **467**, 1088–1090 (2010).

2. D. E. Penman, J. K. Caves Rugenstein, D. E. Ibarra, M. J. Winnick, Silicate weathering as a feedback and forcing in Earth's climate and carbon cycle. *Earth Sci. Rev.* **209**, 103298 (2020).

3. J. D. Milliman, J. P. M. Syvitski, Geomorphic/tectonic control of sediment discharge to the ocean: The importance of small mountainous rivers. *J. Geol.* **100**, 525–544 (1992).

4. W. W. Hay, Detrital sediment fluxes from continents to oceans. *Chem. Geol.* **145**, 287–323 (1998).

5. J. Hartmann, N. Moosdorf, R. Lauerwald, M. Hinderer, A. J. West, Global chemical weathering and associated P-release—The role of lithology, temperature and soil properties. *Chem. Geol.* **363**, 145–163 (2014).

6. A. G. Lipp, O. Shorttle, E. A. Sperling, J. J. Brocks, D. B. Cole, P. W. Crockford, L. Del Mouro, K. Dewing, S. Q. Dornbos, J. F. Emmings, U. C. Farrell, A. Jarrett, B. W. Johnson, P. Kabanov, C. B. Keller, M. Kunzmann, A. J. Miller, N. T. Mills, B. O'Connell, S. E. Peters, N. J. Planavsky, S. R. Ritzer, S. D. Schoepfer, P. R. Wilby, J. Yang, The composition and weathering of the continents over geologic time. *Geochem. Perspect. Lett.* **17**, 21–26 (2020).

7. C.-T. A. Lee, S. Thurner, S. Paterson, W. Cao, The rise and fall of continental arcs: Interplays between magmatism, uplift, weathering, and climate. *Earth Planet. Sci. Lett.* **425**, 105–119 (2015).

8. A. Dielforder, R. Hetzel, O. Oncken, Megathrust shear force controls mountain height at convergent plate margins. *Nature* **582**, 225–229 (2020).

9. P. A. Cawood, C. J. Hawkesworth, Continental crustal volume, thickness and area, and their geodynamic implications. *Gondw. Res.* **66**, 116–125 (2019).

10. M. Tang, H. Chen, C.-T. A. Lee, W., Cao Subaerial crust emergence hindered by phase-driven lower crust densification on early Earth. *Sci. Adv.* **10**, eadq1952 (2024).

11. D. L. Roerdink, Y. Ronen, H. Strauss, P. R. D. Mason, Emergence of felsic crust and subaerial weathering recorded in Palaeoarchaean barite. *Nat. Geosci.* **15**, 227–232 (2022).

12. B. S. Kamber, The evolving nature of terrestrial crust from the Hadean, through the Archaean, into the Proterozoic. *Precambrian Res.* **258**, 48–82 (2015).

13. I. N. Bindeman, D. O. Zakharov, J. Palandri, N. D. Greber, N. Dauphas, G. J. Retallack, A. Hofmann, J. S. Lackey, A. Bekker, Rapid emergence of subaerial landmasses and onset of a modern hydrologic cycle 2.5 billion years ago. *Nature* **557**, 545–548 (2018).

14. M. Brown, T. Johnson, Secular change in metamorphism and the onset of global plate tectonics. *Am. Mineral.* **103**, 181–196 (2018).

15. R. J. Stern, The evolution of plate tectonics. *Phil. Trans. R. Soc. A* **376**, 20170406 (2018).

16. R. M. Palin, M. Santosh, W. Cao, S.-S. Li, D. Hernández-Uribe, A. Parsons, Secular change and the onset of plate tectonics on Earth. *Earth Sci. Rev.* **207**, 103172 (2020).

17. V. Magni, P. Bouilhol, J. van Hunen, Deep water recycling through time. *Geochem. Geophys. Geosyst.* **15**, 4203–4216 (2014).

18. W. Bleeker, The late Archean record: A puzzle in ca. 35 pieces. *Lithos* **71**, 99–134 (2003).

19. R. N. Mitchell, N. Zhang, J. Salminen, Y. Liu, C. J. Spencer, B. Steinberger, J. B. Murphy, Z.-X. Li, The supercontinent cycle. *Nat. Rev. Earth Environ.* **2**, 358–374 (2021).

20. Z.-X. Li, Y. Liu, R. Ernst, A dynamic 2000—540 Ma Earth history: From cratonic amalgamation to the age of supercontinent cycle. *Earth Sci. Rev.* **238**, 104336 (2023).

21. G. A. Shields, A normalised seawater strontium isotope curve: Possible implications for Neoproterozoic-Cambrian weathering rates and the further oxygenation of the Earth. *eEarth* **2**, 35–42 (2007).

22. D. E. Canfield, J. Farquhar, Animal evolution, bioturbation, and the sulfate concentration of the oceans. *Proc. Natl. Acad. Sci. U.S.A.* **106**, 8123–8127 (2009).

23. C. T. Reinhard, N. J. Planavsky, B. C. Gill, K. Ozaki, L. J. Robbins, T. W. Lyons, W. W. Fischer, C. Wang, D. B. Cole, K. O. Konhauser, Evolution of the global phosphorus cycle. *Nature* **541**, 386–389 (2017).

24. D. E. Canfield, A. Teske, Late Proterozoic rise in atmospheric oxygen concentration inferred from phylogenetic and sulphur-isotope studies. *Nature* **382**, 127–132 (1996).

25. J. Farquhar, H. Bao, M. Thiemens, Atmospheric influence of Earth's earliest sulfur cycle. *Science* **289**, 756–758 (2000).

26. A. Bekker, H. D. Holland, P.-L. Wang, D. Rumble III, H. J. Stein, J. L. Hannah, L. L. Coetzee, N. J. Beukes, Dating the rise of atmospheric oxygen. *Nature* **427**, 117–120 (2004).

27. L. M. Och, G. A. Shields-Zhou, The neoproterozoic oxygenation event: Environmental perturbations and biogeochemical cycling. *Earth Sci. Rev.* **110**, 26–57 (2012).

28. T. W. Lyons, C. T. Reinhard, N. J. Planavsky, The rise of oxygen in Earth's early ocean and atmosphere. *Nature* **506**, 307–315 (2014).

29. A. P. Gumsley, K. R. Chamberlain, W. Bleeker, U. Söderlund, M. O. de Kock, E. R. Larsson, A. Bekker, Timing and tempo of the great oxidation event. *Proc. Natl. Acad. Sci. U.S.A.* **114**, 1811–1816 (2017).

30. P. F. Hoffman, A. J. Kaufman, G. P. Halverson, D. P. Schrag, A neoproterozoic snowball earth. *Science* **281**, 1342–1346 (1998).

31. R. E. Kopp, J. L. Kirschvink, I. A. Hilburn, C. Z. Nash, The Paleoproterozoic snowball Earth: A climate disaster triggered by the evolution of oxygenic photosynthesis. *Proc. Natl. Acad. Sci. U.S.A.* **102**, 11131–11136 (2005).

32. A. D. Rooney, J. V. Strauss, A. D. Brandon, F. A. Macdonald, A Cryogenian chronology: Two long-lasting synchronous Neoproterozoic glaciations. *Geology* **43**, 459–462 (2015).

33. J. L. Payne, A. G. Boyer, J. H. Brown, S. Finnegan, M. Kowalewski, R. A. Krause Jr., S. K. Lyons, C. R. McClain, D. W. McShea, P. M. Novack-Gottshall, F. A. Smith, J. A. Stempien, S. C. Wang, Two-phase in-crease in the maximum size of life over 3.5 billion years reflects biological innovation and environmental opportunity. *Proc. Natl. Acad. Sci. U.S.A.* **106**, 24–27 (2009).

34. P. Luffi, M. N. Ducea, Chemical mohometry: Assessing crustal thickness of ancient oro-gens using geochemical and isotopic data. *Rev. Geophys.* **60**, e2021RG000753 (2022).

35. N. M. W. Roberts, C. J. Spencer, S. Puetz, C. B. Keller, S. Tapster, Regional trends and petrologic factors inhibit global interpretations of zircon trace element compositions. *Geosci. Front.* **15**, 101852 (2024).

36. S. Lallemand, A. Heuret, D. Boutelier, On the relationships between slab dip, back-arc stress, upper plate absolute motion, and crustal nature in subduction zones. *Geochem. Geophys. Geosyst.* **6**, Q09006 (2005).

37. D. J. J. van Hinsbergen, T. Schouten, Deciphering paleogeography from orogenic architecture: Constructing orogens in a future supercontinent as thought experiment. *Am. J. Sci.* **321**, 955–1031 (2022).

38. T. Ito, Y. Kojima, S. Kodaira, H. Sato, Y. Kaneda, T. Iwasaki, E. Kurashimo, N. Tsumura, A. Fujiwara, T. Miyauchi, N. Hirata, S. Harder, K. Miller, A. Murata, S. Yamakita, M. Onishi, S. Abe, T. Sato, T. Ikawa, Crustal structure of southwest Japan, revealed by the in-tegrated seismic experiment Southwest Japan 2002. *Tectonophysics* **472**, 124–134 (2009).

39. J. Nábělek, G. Hetényi, J. Vergne, G. Hetényi, J. Vergne, S. Sapkota, B. Kafle, M. Jiang, H. Su, J. Chen, B.-S. Huang, Hi-CLIMB Team, Underplating in the Himalaya-Tibet collision zone revealed by the Hi-CLIMB experiment. *Science* **325**, 1371–1374 (2009).

40. A. R. C. Kylander-Clark, B. R. Hacker, C. G. Mattinson, Size and exhumation rate of ultra-high-pressure terranes linked to orogenic stage. *Earth Planet. Sci. Lett.* **321-322**, 115–120 (2012).

41. E. Burov, T. Francois, P. Yamato, S. Wolf, Mechanisms of continental subduction and exhumation of HP and UHP rocks. *Gondwana Res.* **25**, 464–493 (2014).

42. J. P. Butler, C. Beaumont, R. A. Jamieson, Paradigm lost: Buoyancy thwarted by the strength of the Western Gneiss Region (ultra)high-pressure terrane, Norway. *Lithosphere* **7**, 379–407 (2015).

43. B. R. Hacker, T. B. Andersen, S. Johnston, A. R. C. Kylander-Clark, E. M. Peterman, E. O. Walsh, D. Young, High-temperature deformation during continental-margin subduction & exhumation: The ultrahigh-pressure Western Gneiss Region of Norway. *Tectonophysics* **480**, 149–171 (2010).

44. D. J. J. van Hinsbergen, T. Lamont, C. Guilmette, Lithospheric unzipping explaining hot orogenesis during continental subduction. *Tectonics* **44**, e2025TC008993 (2025).

45. A. Paul, D. Hatzfeld, A. Kaviani, M. Tatar, C. Péquegnat, Seismic imaging of the lithospheric structure of the Zagros mountain belt (Iran). *Geol. Soc. Lond. Spec. Publ.* **330**, 5–18 (2010).

46. S. Lallemand, Y. Font, H. Bijwaard, H. Cao, New insights on 3-D plates interaction near Taiwan from tomography and tectonic implications. *Tectonophysics* **335**, 229–253 (2001).

47. T. J. Owens, G. Zandt, Implications of crustal property variations for models of Tibetan plateau evolution. *Nature* **387**, 37–43 (1997).

48. F. M. Schneider, X. Yuan, B. Schurr, J. Mechie, C. Sippl, S.-K. Kufner, L. Ratschbacher, F. Tilmann, I. Oimahmadov, M. Gadoev, V. Minaev, U. Abdybachaev, S. Orunbaev, A. Ischuk, S. Murodkulov, The crust in the Pamir: Insights from receiver functions. *J. Geophys. Res. Solid Earth* **124**, 9313–9331 (2019).

49. M. Brown, T. Johnson, Time's arrow, time's cycle: Granulite metamorphism and geodynamics. *Mineral. Mag.* **83**, 323–338 (2019).

50. A. Brudner, H. Jiang, X. Chu, M. Tang, Crustal thickness of the Grenville orogen: A mesoproterozoic Tibet? *Geology* **50**, 402–406 (2021).

51. I. W. Hillenbrand, A. K. Gilmer, M. L. Williams, K. E. Karlstrom, A. K. Souders, M. J. Jercinovic, J. Vazquez, W. R. Premo, Heterogeneous multi-stage accretionary orogenesis—Evidence from the Gunnison block in the Yavapai Province, southwest USA. *Precambrian Res.* **401**, 107256 (2024).

52. D. Corrigan, S. Pehrsson, N. Wodicka, E. de Kemp, The palaeoproterozoic trans-hudson orogen: A prototype of modern accretionary processes. *Geol. Soc. Lond. Spec. Publ.* **327**, 457–479 (2009).

53. A. Gilligan, I. D. Bastow, F. A. Darbyshire, Seismological structure of the 1.8 Ga Trans-Hudson Orogen of North America. *Geochem. Geophys. Geosyst.* **17**, 2421–2433 (2016).

54. R. M. Palin, R. W. White, E. C. R. Green, Partial melting of metabasic rocks and the generation of tonalitic–trondhjemitic–granodioritic (TTG) crust in the Archaean: Constraints from phase equilibrium modelling. *Precambrian Res.* **287**, 73–90 (2016).

55. M. A. Smit, K. A. Musiyachenko, J. Goumans, Archaean continental crust formed from mafic cumulates. *Nat. Commun.* **15**, 692 (2024).

56. C. Herzberg, R. Rudnick, Formation of cratonic lithosphere: An integrated thermal and petrological model. *Lithos* **149**, 4–15 (2012).

57. T. E. Johnson, M. Brown, B. J. P. Kaus, J. A. VanTongeren, Delamination and recycling of Archaean crust caused by gravitational instabilities. *Nat. Geosci.* **7**, 47–52 (2013).

58. G. Toussaint, E. Burov, L. Jolivet, Continental plate collision: Unstable vs. stable slab dynamics. *Geology* **32**, 33 (2004).

59. N. I. Christensen, W. D. Mooney, Seismic velocity structure and composition of the continental crust: A global view. *J. Geophys. Res. Solid Earth* **100**, 9761–9788 (1995).

60. M. J. Unsworth, A. G. Jones, W. Wei, G. Marquis, S. G. Gokarn, J. E. Spratt, Crustal rheology of the Himalaya and Southern Tibet inferred from magnetotelluric data. *Nature* **438**, 78–81 (2005).

61. R. M. Palin, J. D. P. Moore, Z. Zhang, G. Huang, J. Wade, B. Dyck, Mafic Archean continental crust prohibited exhumation of orogenic UHP eclogite. *Geosci. Front.* **12**, 101225 (2021).

62. M. Tang, K. Chen, R. L. Rudnick, Archean upper crust transition from mafic to felsic marks the onset of plate tectonics. *Science* **351**, 372–375 (2016).

63. M. A. Smit, K. Mezger, Earth's early O_2 cycle suppressed by primitive continents. *Nat. Geosci.* **10**, 788–792 (2017).

64. D. G. van der Meer, C. R. Scotese, B. J. W. Mills, A. Sluijs, A.-P. van den Berg van Saparoea, R. M. B. van de Weg, Long-term Phanerozoic global mean sea level: Insights from strontium isotope variations and estimates of continental glaciation. *Gondwana Res.* **111**, 103–121 (2022).

65. C. Herzberg, K. Condie, J. Korenaga, Thermal history of the Earth and its petrological expression. *Earth Planet. Sci. Lett.* **292**, 79–88 (2010).

66. D. C. Bradley, Passive margins through earth history. *Earth Sci. Rev.* **91**, 1–26 (2008).

67. M. A. Kipp, E. E. Stüeken, Biomass recycling and Earth's early phosphorus cycle. *Sci. Adv.* **3**, eaao4795 (2017).

68. H. E. Hartnett, R. G. Keil, J. I. Hedges, A. H. Devol, Influence of oxygen exposure time on organic carbon preservation in continental margin sediments. *Nature* **391**, 572–575 (1998).

69. J. R. Zondervan, R. G. Hilton, M. Dellinger, F. J. Clubb, T. Roylands, M. Ogrič, Rock organic carbon oxidation CO_2 release offsets silicate weathering sink. *Nature* **623**, 329–333 (2023).

70. E. E. Scherer, K. L. Cameron, J. Blichert-Toft, Lu–Hf garnet geochronology: Closure temperature relative to the Sm–Nd system and the effects of trace mineral inclusions. *Geochim. Cosmochim. Acta* **64**, 3413–3432 (2000).

71. E. F. Baxter, E. E. Scherer, Garnet geochronology: Timekeeper of tectonometamorphic processes. *Elements* **9**, 433–438 (2013).

72. M. A. Smit, J. C. Vrijmoed, E. E. Scherer, K. Mezger, E. Kooijman, M. Schmitt-Kielman, L. Tual, C. Guilmette, L. Ratschbacher, Retentiveness of rare earth elements in garnet with implications for garnet Lu-Hf chronology. *J. Metam. Geol.* **42**, 703–727 (2024).

73. H. L. Yu, L. F. Zhang, C. J. Wei, X. L. Li, J. H. Guo, Age and P–T conditions of the Gridino-type eclogite in the Belomorian Province, Russia. *J. Metamorph. Geol.* **35**, 855–869 (2017).

74. J. A. Cutts, D. Regis, S. J. Pehrsson, R. Graziani, D. C. Petts, M. A. Smit, B. Knox, Circa 1.9 Ga Rae-Hearne collision from Lu–Hf garnet chronology in eclogites. *Terra Nova* **36**, 284–291 (2024).

75. A. Godet, M. Jouvent, C. Laureijs, C. Guilmette, K. Larson, M. Coleman, J. Darveau, M. Côté-Roberge, In-situ Lu-Hf garnet dating of Archean deep crust granulites from the polymetamorphic Grenville Front Tectonic Zone. *J. Petrol.* **65**, egae080 (2024).

76. Y.-Q. Yan, H. Wang, J.-H. Yang, S.-T. Wu, J. Ran, Reevaluating Archean eclogite-facies metamorphism in the North China Craton. *Geology* **54**, 425–429 (2026).

77. R. Yang, H. Cheng, G. Limin, K.-Y. Du, Q. Chen, Accurate dating of metamorphism requires multiple chronometers: Evidence against Archean modern-style plate tectonics in the North China Craton. *Geology* **54**, 631–635 (2026).

78. A. Simpson, S. Gilbert, R. Tamblyn, M. Hand, C. Spandler, J. Gillespie, A. Nixon, S. Glorie, In-situ Lu Hf geochronology of garnet, apatite and xenotime by LA ICP MS/MS. *Chem. Geol.* **577**, 120299 (2021).

79. L. J. Millonig, R. Albert, A. Gerdes, D. Avigad, C. Dietsch, Exploring laser ablation U–Pb dating of regional metamorphic garnet–The Straits Schist, Connecticut, USA. *Earth Planet. Sci. Lett.* **552**, 116589 (2020).

80. R. W. Kay, S. Mahlburg Kay, Delamination and delamination magmatism. *Tectonophysics* **219**, 177–189 (1993).

81. E. Kooijman, M. A. Smit, L. Ratschbacher, A. R. C. Kylander-Clark, A view into crustal evolution at mantle depths. *Earth Planet. Sci. Lett.* **465**, 59–69 (2017).

82. S. M. Schmalholz, Y. Podladchikov, Metamorphism under stress: The problem of relating minerals to depth. *Geology* **42**, 733–734 (2014).

83. E. Moulas, S. M. Schmalholz, Y. Podladchikov, L. Tajčmanová, D. Kostopoulos, L. Baumgartner, Relation between mean stress, thermodynamic, and lithostatic pressure. *J. Metam. Geol.* **37**, 1–14 (2019).

84. A. Bauville, P. Yamato, Pressure-to-depth conversion models for metamorphic rocks: Derivation and applications. *Geochem. Geophys. Geosyst.* **22**, e2020GC009280 (2021).

85. R. M. Holder, D. R. Viete, M. Brown, T. E. Johnson, Metamorphism and the evolution of plate tectonics. *Nature* **572**, 378–381 (2019).

86. J. Kuang, G. Morra, D. A. Yuen, T. Kusky, S. Jiang, H. Yao, S. Qi, Metamorphic constraints on Archean tectonics. *Precambrian Res.* **397**, 107195 (2023).

87. D. Loose, V. Schenk, 2.09 Ga old eclogites in the Eburnean–Trans Amazonian orogeny of southern Cameroon: Significance for a Paleoproterozoic plate tectonics. *Precambrian Res.* **304**, 1–11 (2018).

88. C. François, V. Debaille, J.-L. Paquette, D. Baudet, E. J. Javaux, The earliest evidence for modern-style plate tectonics recorded by HP–LT metamorphism in the Paleoproterozoic of the Democratic Republic of the Congo. *Sci. Rep.* **8**, 15452 (2018).

89. J. Blichert-Toft, R. Frei, Complex Sm-Nd and Lu-Hf isotope systematics in metamorphic garnets from the Isua supracrustal belt, West Greenland. *Geochim. Cosmochim. Acta* **65**, 3177–3189 (2001).

90. B. Eskesen, K. Fassmer, C. Münker, T. Ulrich, K. Szilas, S. Wagner, J. E. Hoffmann, T. J. Nagel, Neoarchean synkinematic metamorphic peak in the Isua supracrustal belt (West Greenland). *Geology* **51**, 1017–1021 (2023).

91. J. Alfing, T. E. Johnson, J. Kaempf, M. Brown, K. Szilas, K. Rankenburg, C. Clark, Eoarchean granulite-facies metamorphism in the Itsaq Gneiss Complex, southwest Greenland. *Earth Planet. Sci. Lett.* **646**, 118977 (2024).

92. J. Kaempf, T. E. Johnson, C. Clark, J. Alfing, M. Brown, P. Lanari, K. Rankenburg, Paleoarchean metamorphism in the Acasta Gneiss Complex: Constraints from phase equilibrium modelling and in situ garnet Lu–Hf geochronology. *J. Metam. Geol.* **42**, 373–394 (2024).

93. K. A. Cutts, G. Stevens, J. E. Hoffmann, I. S. Buick, D. Frei, C. Münker, Paleo- to mesoarchean polymetamorphism in the Barberton Granite-Greenstone Belt, South Africa: Constraints from U-Pb monazite and Lu-Hf garnet geochronology on the tectonic processes that shaped the belt. *Geol. Soc. Am. Bull.* **126**, 251–270 (2013).

94. M. V. Mints, E. A. Belousova, A. N. Konilov, L. M. Natapov, A. A. Shchipansky, W. L. Griffin, S. Y. O'Reilly, K. A. Dokukina, T. V. Kaulina, Mesoarchean subduction processes: 2.87 Ga eclogites from the Kola Peninsula, Russia. *Geology* **38**, 739–742 (2010).

95. D. Herwartz, S. G. Skublov, A. V. Berezin, A. E. Mel'nik, First Lu-Hf garnet ages of eclogites from the Belomorian Mobile Belt (Baltic Shield, Russia). *Dokl. Earth Sci.* **443**, 377–380 (2012).

96. M. Hand, D. Brown, S. Glorie, X. He, S. Gilbert, J. Payne, "In-situ garnet Lu-Hf geochronology from the contentious Belomorian high-pressure rocks," (Copernicus GmbH, 2025); https://doi.org/10.5194/egusphere-egu24-4969.

97. W. Ning, T. Kusky, L. Wang, B. Huang, Archean eclogite-facies oceanic crust indicates modern-style plate tectonics. *Proc. Natl. Acad. Sci. U.S.A.* **119**, e2117529119 (2022).

98. M. Huang, S. Jiao, T. E. Johnson, C. Clark, J. Yu, G. Huang, J. Guo, Eclogite-facies metamorphism of continental crust at the Mesoarchean-to-Neoarchean transition. *Geology* **54**, 269–274 (2026).

99. T. E. Johnson, R. W. White, Phase equilibrium constraints on conditions of granulite-facies metamorphism at Scourie, NW Scotland. *J. Geol. Soc. London* **168**, 147–158 (2011).

100. Y. Feisel, R. W. White, R. M. Palin, T. E. Johnson, New constraints on granulite facies metamorphism and melt production in the Lewisian Complex, northwest Scotland. *J. Metam. Geol.* **36**, 799–819 (2018).

101. P. Gopon, J. B. Forshaw, J. Wade, D. J. Waters, C. Gopon, Seeing through metamorphic overprints in Archean granulites: Combined high-resolution thermometry and phase equilibrium modeling of the Lewisian Complex, Scotland. *Am. Min.* **107**, 1487–1500 (2022).

102. S. R. Miocevich, A. Copley, O. M. Weller, Testing the importance of sagduction: Insights from the Lewisian Gneiss Complex of northwest Scotland. *Precambrian Res.* **379**, 106708 (2022).

103. V. A. Kamzolkin, S. D. Ivanov, A. N. Konilov, Empirical phengite geobarometer: Background, calibration, and application. *Geol. Ore Deposits* **58**, 613–622 (2016).

104. R. Tamblyn, D. Brown, M. Hand, L. Morrissey, C. Clark, R. Anczkiewicz, The 2 Ga eclogites of Central Tanzania: Directly linking age and metamorphism. *Lithos* **380-381**, 105890 (2021).

105. O. M. Weller, M. R. St-Onge, Record of modern-style plate tectonics in the Palaeoproterozoic Trans-Hudson orogen. *Nat. Geosci.* **10**, 305–311 (2017).

106. W. D. Carlson, S. D. Anderson, S. Mosher, J. S. Davidow, W. D. Crawford, E. D. Lane, High-pressure metamorphism in the Texas Grenville Orogen: Mesoproterozoic subduction of the southern laurentian continental margin. *Int. Geol. Rev.* **49**, 99–119 (2007).

107. J. S. F. Levine, S. Mosher, Contrasting grenville-aged tectonic histories across the Llano Uplift, Texas: New evidence for deep-seated high-temperature deformation in the western uplift. *Lithosphere* **2**, 399–410 (2010).

108. A. Bird, M. Thirlwall, R. Strachan, I. Millar, E. Dempsey, K. Hardman, Eclogites and basement terrane tectonics in the northern arm of the Grenville orogen, NW Scotland. *Geosci. Front.* **14**, 101668 (2023).

109. L. Tual, C. Möller, M. J. Whitehouse, Tracking the prograde P–T path of Precambrian eclogite using Ti-in-quartz and Zr-in-rutile geothermobarometry. *Contrib. Mineral. Petrol.* **173**, 56 (2018).

110. C. Möller, J. Andersson, B. Dyck, I. A. Lundin, Exhumation of an eclogite terrane as a hot migmatitic nappe, Sveconorwegian orogen. *Lithos* **226**, 147–168 (2015).

111. J. P. Liégeois, L. Latouche, M. Boughrara, J. Navez, M. Guiraud, The LATEA metacraton (Central Hoggar, Tuareg shield, Algeria): Behaviour of an old passive margin during the Pan-African orogeny. *J. Afr. Earth Sci.* **37**, 161–190 (2003).

112. S. A. Doukkari, K. Ouzegane, A. Arab, J.-R. Kienast, G. Godard, A. Drareni, S. Zetoutou, J.-P. Liégeois, Phase relationships and P–T path in NCFMASHTO system of the eclogite from the Tighsi area (Egere terrane, Central Hoggar, Algeria). *J. Afr. Earth Sci.* **99**, 276–286 (2014).

113. J. J. Peucat, Y. Ohta, D. G. Gee, J. Bernard-Griffiths, U-Pb, Sr and Nd evidence for grenvillian and latest proterozoic tectonothermal activity in the spitsbergen caledonides, arctic ocean. *Lithos* **22**, 275–285 (1989).

114. R. D. Dallmeyer, J. J. Peucat, T. Hirajima, Y. Ohta, Tectonothermal chronology within a blueschist-eclogite complex, west-central Spitsbergen, Svalbard: Evidence from $^{40}Ar/^{39}Ar$ and Rb-Sr mineral ages. *Lithos* **24**, 291–304 (1990).

115. S. Elvevold, E. J. K. Ravna, P. Nasipuri, L. Labrousse, Calculated phase equilibria for phengite-bearing eclogites from NW Spitsbergen, Svalbard Caledonides. *Geol. Soc. Lond. Spec. Publ.* **390**, 385–401 (2013).

116. T. J. S. dos Santos, M. da G. M. Garcia, W. S. Amaral, R. Caby, E. Wernick, M. H. Arthaud, E. L. Dantas, M. Santosh, Relics of eclogite facies assemblages in the Ceará Central

Domain, NW Borborema Province, NE Brazil: Implications for the assembly of West Gondwana. *Gondwana Res.* **15**, 454–470 (2009).

117. C. E. Ganade de Araujo, R. F. Weinberg, U. G. Cordani, Extruding the Borborema Province (NE-Brazil): A two-stage Neoproterozoic collision process. *Terra Nova* **26**, 157–168 (2014).

118. T. J. S. dos Santos, W. da S. Amaral, M. F. Ancelmi, M. Z. Pitarello, R. A. Fuck, E. L. Dantas, U–Pb age of the coesite-bearing eclogite from NW Borborema Province, NE Brazil: Implications for western Gondwana assembly. *Gondwana Res.* **28**, 1183–1196 (2015).

119. C. Zhang, L. Zhang, H. V. Roermund, S. Song, G. Zhang, Petrology and SHRIMP U–Pb dating of Xitieshan eclogite, North Qaidam UHP metamorphic belt, NW China. *J. Asian Earth Sci.* **42**, 752–767 (2011).

120. S. Song, Y. Niu, L. Su, C. Zhang, L. Zhang, Continental orogenesis from ocean subduction, continent collision/subduction, to orogen collapse, and orogen recycling: The example of the North Qaidam UHPM belt, NW China. *Earth Sci. Rev.* **129**, 59–84 (2014).

121. D. A. Carswell, R. D. Tucker, P. J. O'Brien, T. E. Krogh, Coesite micro-inclusions and the U/Pb age of zircons from the Hareidland Eclogite in the Western Gneiss Region of Norway. *Lithos* **67**, 181–190 (2003).

122. J. A. Cutts, M. A. Smit, Rates of deep continental burial from Lu-Hf garnet chronology and Zr-in-Rutile thermometry on (ultra)high-pressure rocks. *Tectonics* **37**, 71–88 (2018).

123. C. G. Mattinson, R. Y. Zhang, T. Tsujimori, J. G. Liou, Epidote-rich talc-kyanite-phengite eclogites, Sulu terrane, Eastern China: P-T-fO_2 estimates and the significance of the epidote-talc assemblage in eclogite. *Am. Mineral.* **89**, 1772–1783 (2004).

124. K. Zong, Y. Liu, Z. Hu, T. Kusky, D. Wang, C. Gao, S. Gao, J. Wang, Melting-induced fluid flow during exhumation of gneisses of the Sulu ultrahigh-pressure terrane. *Lithos* **120**, 490–510 (2010).

125. S. L. Baldwin, B. D. Monteleone, L. E. Webb, P. G. Fitzgerald, M. Grove, E. June Hill, Pliocene eclogite exhumation at plate tectonic rates in eastern Papua New Guinea. *Nature* **431**, 263–267 (2004).

126. J. W. DesOrmeau, S. M. Gordon, T. A. Little, S. A. Bowring, B. Schoene, K. M. Samperton, A. R. C. Kylander-Clark, Using eclogite retrogression to track the rapid exhumation of the Pliocene Papua New Guinea UHP Terrane. *J. Petrol.* **59**, 2017–2042 (2018).

127. G. Wang, H. Thybo, I. M. Artemieva, No mafic layer in 80 km thick Tibetan crust. *Nat. Commun.* **12**, 1069 (2021).

128. D. E. Canfield, A new model for Proterozoic ocean chemistry. *Nature* **396**, 450–453 (1998).

129. A. D. Anbar, Y. Duan, T. W. Lyons, G. L. Arnold, B. Kendall, R. A. Creaser, A. J. Kaufman, G. W. Gordon, C. Scott, J. Garvin, R. Buick, A whiff of oxygen before the great oxidation event? *Science* **317**, 1903–1906 (2007).

130. Y. Duan, A. D. Anbar, G. L. Arnold, T. W. Lyons, G. W. Gordon, B. Kendall, Molybdenum isotope evidence for mild environmental oxygenation before the Great Oxidation Event. *Geochim. Cosmochim. Acta* **74**, 6655–6668 (2010).

131. S. A. Crowe, L. N. Døssing, N. J. Beukes, M. Bau, S. J. Kruger, R. Frei, D. E. Canfield, Atmospheric oxygenation three billion years ago. *Nature* **501**, 535–538 (2013).

132. K. V. Lau, F. A. Macdonald, K. Maher, J. L. Payne, Uranium isotope evidence for temporary ocean oxygenation in the aftermath of the Sturtian Snowball Earth. *Earth Planet. Sci. Lett.* **458**, 282–292 (2017).

133. G. Avice, B. Marty, R. Burgess, A. Hofmann, P. Philippot, K. Zahnle, D. Zakharov, Evolution of atmospheric xenon and other noble gases inferred from Archean to Paleoproterozoic rocks. *Geochim. Cosmochim. Acta* **232**, 82–100 (2018).

134. D. C. Catling, K. J. Zahnle, C. P. McKay, Biogenic methane, hydrogen escape, and the irreversible oxidation of early Earth. *Science* **293**, 839–843 (2001).

135. H. D. Holland, Volcanic gases, black smokers, and the great oxidation event. *Geochim. Cosmochim. Acta* **66**, 3811–3826 (2002).

136. L. R. Kump, M. E. Barley, Increased subaerial volcanism and the rise of atmospheric oxygen 2.5 billion years ago. *Nature* **448**, 1033–1036 (2007).

137. C.-T. A. Lee, L. Y. Yeung, N. R. McKenzie, Y. Yokoyama, K. Ozaki, A. Lenardic, Two-step rise of atmospheric oxygen linked to the growth of continents. *Nat. Geosci.* **9**, 417–424 (2016).

138. D. C. Catling, K. J. Zahnle, The Archean atmosphere. *Sci. Adv.* **6**, eaax1420 (2020).

139. D. A. Fike, A. S. Bradley, C. V. Rose, Rethinking the ancient sulfur cycle. *Annu. Rev. Earth Planet. Sci.* **43**, 593–622 (2015).

140. D. E. Canfield, The early history of atmospheric oxygen: Homage to Robert M. Garrels. *Annu. Rev. Earth Planet. Sci.* **33**, 1–36 (2005).

141. A. Bachan, L. R. Kump, The rise of oxygen and siderite oxidation during the Lomagundi Event. *Proc. Natl. Acad. Sci. U.S.A.* **112**, 6562–6567 (2015).

142. A. P. Martin, D. J. Condon, A. R. Prave, A. Lepland, A review of temporal constraints for the Palaeoproterozoic large, positive carbonate carbon isotope excursion (the Lomagundi–Jatuli Event). *Earth Sci. Rev.* **127**, 242–261 (2013).

143. M. R. Warke, T. Di Rocco, A. L. Zerkle, A. Lepland, A. R. Prave, A. P. Martin, Y. Ueno, D. J. Condon, M. W. Claire, The great oxidation event preceded a Paleoproterozoic "snowball Earth". *Proc. Natl. Acad. Sci.* **117**, 13314–13320 (2020).

144. M. S. W. Hodgskiss, P. W. Crockford, A. V. Turchyn, Deconstructing the lomagundi-jatuli carbon isotope excursion. *Annu. Rev. Earth Planet. Sci.* **51**, 301–330 (2023).

145. K. O. Konhauser, E. Pecoits, S. V. Lalonde, D. Papineau, E. G. Nisbet, M. E. Barley, N. T. Arndt, K. Zahnle, B. S. Kamber, Oceanic nickel depletion and a methanogen famine before the Great Oxidation Event. *Nature* **458**, 750–753 (2009).

146. J. Kirschvink, "Late Proterozoic low-latitude global glaciation: The Snowball Earth" in *The Proterozoic Biosphere: A Multidisciplinary Study* (Cambridge Univ. Press, 1992), pp. 51–52.

147. K. C. Condie, E. Beyer, E. Belousova, W. L. Griffin, S. Y. O'Reilly, U–Pb isotopic ages and Hf isotopic composition of single zircons: The search for juvenile Precambrian continental crust. *Precambrian Res.* **139**, 42–100 (2005).

148. E. Sizova, T. Gerya, K. Stüwe, M. Brown, Generation of felsic crust in the Archean: A geodynamic modeling perspective. *Precambrian Res.* **271**, 198–224 (2015).

149. J. van Hunen, J.-F. Moyen, Archean subduction: Fact or fiction? *Annu. Rev. Earth Planet. Sci.* **40**, 195–219 (2012).

150. O. Laurent, H. Martin, J. F. Moyen, R. Doucelance, The diversity and evolution of late-Archean granitoids: Evidence for the onset of "modern-style" plate tectonics between 3.0 and 2.5 Ga. *Lithos* **205**, 208–235 (2014).

151. T. Gerya, Precambrian geodynamics: Concepts and models. *Gondwana Res.* **25**, 442–463 (2014).

152. J. H. Bédard, A catalytic delamination-driven model for coupled genesis of Archaean crust and sub-continental lithospheric mantle. *Geochim. Cosmochim. Acta* **70**, 1188–1214 (2006).

153. O. Nebel, F. A. Capitanio, J.-F. Moyen, R. F. Weinberg, F. Clos, Y. J. Nebel-Jacobsen, P. A. Cawood, When crust comes of age: On the chemical evolution of Archaean, felsic continental crust by crustal drip tectonics. *Philos. Trans. A Math. Phys. Eng. Sci.* **376**, 20180103 (2018).

154. M. Brown, C. L. Kirkland, T. E. Johnson, Evolution of geodynamics since the Archean: Significant change at the dawn of the Phanerozoic. *Geology* **48**, 488–492 (2020).

155. P. A. Cawood, Earth Matters: A tempo to our planet's evolution. *Geology* **48**, 525–526 (2020).

156. R. H. Smithies, D. C. Champion, M. J. Van Kranendonk, Formation of Paleoarchean continental crust through infracrustal melting of enriched basalt. *Earth Planet. Sci. Lett.* **281**, 298–306 (2009).

157. M. A. Smit, A. Scherstén, T. Næraa, R. B. Emo, E. E. Scherer, P. Sprung, W. Bleeker, K. Mezger, A. Maltese, Y. Cai, E. T. Rasbury, M. J. Whitehouse, Formation of Archean continental crust constrained by boron isotopes. *Geochem. Perspect. Lett.* **12**, 23–26 (2019).

158. J. Halla, The TTG-amphibolite terrains of arctic fennoscandia: Infinite networks of amphibolite metatexite-diatexite transitions. *Front. Earth Sci.* **8**, 252 (2020).

159. S. B. Shirey, S. H. Richardson, Start of the Wilson cycle at 3 Ga shown by diamonds from subcontinental mantle. *Science* **333**, 434–436 (2011).

160. A. R. Brenner, R. R. Fu, D. A. D. Evans, A. V. Smirnov, R. Trubko, I. R. Rose, Paleomagnetic evidence for modern-like plate motion velocities at 3.2 Ga. *Sci. Adv.* **6**, eaaz8670 (2020).

161. J. J. W. Rogers, M. Santosh, Configuration of columbia, a mesoproterozoic supercontinent. *Gondwana Res.* **5**, 5–22 (2002).

162. Z. X. Li, R. N. Mitchell, C. J. Spencer, R. Ernst, S. Pisarevsky, U. Kirscher, J. B. Murphy, Decoding Earth's rhythms: Modulation of supercontinent cycles by longer superocean episodes. *Precambrian Res.* **323**, 1–5 (2019).

163. D. A. D. Evans, Z. X. Li, J. B. Murphy, Four-dimensional context of Earth's supercontinents. *Geol. Soc. Lond. Spec. Publ.* **424**, 1–14 (2016).

164. N. R. McKenzie, B. K. Horton, S. E. Loomis, D. F. Stockli, N. J. Planavsky, C.-T. A. Lee, Continental arc volcanism as the principal driver of icehouse-greenhouse variability. *Science* **352**, 444–447 (2016).

165. I. H. Campbell, C. M. Allen, Formation of supercontinents linked to increases in atmospheric oxygen. *Nat. Geosci.* **1**, 554–558 (2008).

166. F. Horton, Did phosphorus derived from the weathering of large igneous provinces fertilize the Neoproterozoic ocean? *Geochem. Geophys. Geosyst.* **16**, 1723–1738 (2015).
